# Early versus interval appendectomy in children with complicated appendicitis: effects on hospital stay and occurrence of severe complications

**DOI:** 10.3389/fped.2026.1788720

**Published:** 2026-03-12

**Authors:** Yannick Schmidt, Franziska Cramer, Oliver Muensterer, Danielle S. Wendling-Keim

**Affiliations:** Department of Pediatric Surgery, Dr. von Hauner Children's Hospital, LMU University Hospital, LMU Munich, Munich, Germany

**Keywords:** complicated appendicitis, early appendectomy, hospital stay, interval appendectomy, pediatric surgery, postoperative complications

## Abstract

**Purpose:**

Acute appendicitis is a common pediatric emergency, yet optimal management of complicated cases remains debated. This study compares outcomes of early vs. interval appendectomy in children with perforated appendicitis.

**Methods:**

A retrospective review of 254 patients (<18 years) treated between January 2012 and December 2023 was conducted. Twenty-two underwent interval appendectomy, and 232 underwent early appendectomy. Demographic and clinical data were analyzed using SPSS v29.0.1.0, with statistical significance defined as *p* < 0.05.

**Results:**

Early appendectomy was associated with a significantly shorter cumulative hospital stay (mean 9.2 days) than interval appendectomy (mean 22.5 days; *p* < 0.001). Overall complication rates were higher in the interval group (10/22) compared to the early group (31/232; *p* < 0.001). However, severe complications, such as ileostomy creation, stump insufficiency, and ileocecal pole resection, occurred exclusively in the early appendectomy group.

**Conclusion:**

Early appendectomy in children with complicated appendicitis results in shorter hospitalization and fewer overall complications but carries a risk of more severe postoperative events. Prospective studies are warranted to refine patient selection and optimize treatment strategies.

## Introduction

Acute complicated appendicitis is one of the most frequent surgical emergencies in children. Current treatment approaches include early appendectomy (EA), initial treatment with intravenous (i.v.) antibiotics followed by interval appendectomy (IA), and exclusive non-operative treatment with i.v. antibiotics.

In current literature, there is no clear consensus regarding the optimal surgical treatment of complicated appendicitis in children. While several authors advocate for EA, citing reduced overall complications and shorter hospital stay, other studies have reported IA as both, safe and effective.

The American Pediatric Surgery Association recommends EA as the preferred strategy for acutely ill children diagnosed with complicated appendicitis, as well as in the absence of abscess formation (1). However, supporters of IA point out that it avoids surgery on inflamed tissues (2). In contrast, Huerta et al. found no relevant differences in the type of complications between IA and EA, whereas the therapy was longer within IA (3).

The identification of prognostic markers may improve treatment decisions. Elevated white blood cell count (WBC), C-reactive protein (CRP) levels and their combined use provides a high sensitivity for diagnosing acute appendicitis (4, 5) In cases of perforation, specificity is particularly high (6). Hyponatremia has also been described as a predictor of perforation in children (7). In addition, fever has been associated with disease severity (8, 9). Younger children represent an especially vulnerable group, as delayed diagnosis is linked to higher complication rates, increased risk of perforation, and prolonged hospitalization (8, 10).

This retrospective, single-center study compares differences in outcomes between the first two options and investigates prognostic factors and initial laboratory parameters including CRP, WBC, serum sodium, body temperature, and age at admission in children that may help guide in choosing the optimal individualized therapeutic approach.

## Patients/materials and methods

### Study design and setting

We conducted a retrospective, observational, single-center study at the department of pediatric surgery of our tertiary care center. The study period spanned 12 years, from January 1, 2012, to December 31, 2023. Patients were identified through the hospital's electronic medical records using ICD-10 codes K36, K35.30, K35.8, K35.32, K35.2, and K35.31.

### Inclusion and exclusion criteria

Children and adolescents younger than 18 years with a diagnosis of complicated appendicitis were eligible for inclusion. Patients entered the study if they underwent either early appendectomy (EA) during the index hospitalization or interval appendectomy (IA) following initial non-operative management.

In the EA group, the diagnosis of complicated appendicitis was defined intraoperatively as perforated appendicitis and subsequently verified by histopathological examination. In the IA group, complicated appendicitis was determined based on the clinical and radiologic diagnosis of a periappendiceal abscess at the initial presentation and further corroborated intraoperatively at the time of IA and on histopathology. Histologic features confirming prior perforation included necrosis, granulation tissue, and fibrosis, consistent with prior descriptions in the literature (11, 12).

Exclusion criteria comprised patients older than 18 years, those with non-complicated appendicitis, appendiceal neoplasms, incomplete medical records, prior abdominal surgery involving the appendix, or transfer to or from an external institution during treatment, which precluded complete assessment of hospital length of stay or outcomes.

Patients were stratified into two groups according to the treatment strategy: early appendectomy (EA) and interval appendectomy (IA). Admission data, including age, sex, C-reactive protein (CRP), white blood cell (WBC) count, serum sodium, and body temperature, were collected and compared between groups.

The primary outcome was defined as the cumulative length of hospital stay (LOH), including all admissions related to appendicitis for the individual patient. Secondary outcomes included the overall complication rate and the type and severity of complications.

### Procedures

A total of 232 patients were treated with early appendectomy and 22 patients underwent interval appendectomy. The treatment strategy was determined at the time of presentation based on shared decision-making between caregivers and treating physicians.

In the EA group, appendectomy was performed laparoscopically according to institutional standards. Perioperatively, patients received intravenous antibiotics, initially consisting of metronidazole in combination with cefotaxime or piperacillin/tazobactam.

In the IA group, patients were initially managed non-operatively with intravenous antibiotic therapy (piperacillin/tazobactam or escalation to meropenem if clinically indicated). CT-guided abscess drainage was performed when necessary. Elective appendectomy was scheduled 6–8 weeks after resolution of the acute inflammatory process. Antibiotic regimens were adapted according to microbiological culture results when abscess drainage was performed. Following discharge from the initial hospitalization, oral antibiotic therapy with amoxicillin/clavulanic acid or metronidazole was prescribed based on the results of the antibiogram, if available. Non-operative treatment was not offered for suspected perforated appendicitis in our institution during the study period.

### Complications

Detection of seroma as well as paralytic ileus, free fluid and generalized peritonitis were included in the complications analysis if they occurred on postoperative day 5 or later after primary surgery.

Seroma was defined as a postoperative sonographically evident, localized fluid collection at the operative site on postoperative day 5 or later.

Paralytic ileus was noted in cases of prolonged postoperative ileus with failure of return of bowel function beyond postoperative day 5, accompanied by abdominal distension, vomiting, and absence of flatus or stool on clinical and, where indicated, radiologic assessment. Events occurring earlier than day 5 were not noted as complications but as part of routine postoperative recovery.

Further, in our analysis, “free fluid” and “generalized peritonitis” were only recorded as postoperative complications when they were detected from postoperative day 5 onwards. Any free fluid or peritonitis present earlier was not counted as a postoperative adverse event.

The complications were categorized using the Clavien-Dindo classification retrospectively.

### Statistical analysis

We analyzed a possible correlation between parameters taken at admission and the length of the hospital stay as well as the complication rate. Comparisons between EA and IA were performed using parametric or non-parametric tests based on data distribution. Age was analyzed with an unpaired t-test, while gender distribution was assessed with Fisher's exact test. Baseline laboratory and clinical parameters, including C-reactive protein (CRP), white blood cell (WBC) count, serum sodium, and body temperature, were compared using the Mann–Whitney U test. To evaluate the association between admission variables (CRP, WBC count, serum sodium, body temperature, and age) and the length of hospitalization (LOH), simple linear regression models were constructed independent of the surgical approach. Complication rates were initially compared using the Mann–Whitney U test. To further examine predictors of postoperative complications, logistic regression analysis was performed with admission variables (CRP, WBC count, serum sodium, body temperature, and age) entered as independent variables.

Given the retrospective design, a formal *a priori* sample size calculation was not feasible. However, *post hoc* power analysis demonstrated that the available cohort provided sufficient power to detect clinically relevant differences in cumulative hospital length of stay between the EA and IA groups.

All statistical analyses were conducted with SPSS version 29.0.1.0 (IBM Corp., Armonk, NY, USA). A two-tailed *p*-value < 0.05 was considered statistically significant.

### Ethical considerations

The study was approved by the institutional ethics committee (approval number: 22-0189). Due to the retrospective nature of the study, the requirement for informed consent was waived in accordance with national regulations and institutional policy.

## Results

**Figure 1:**
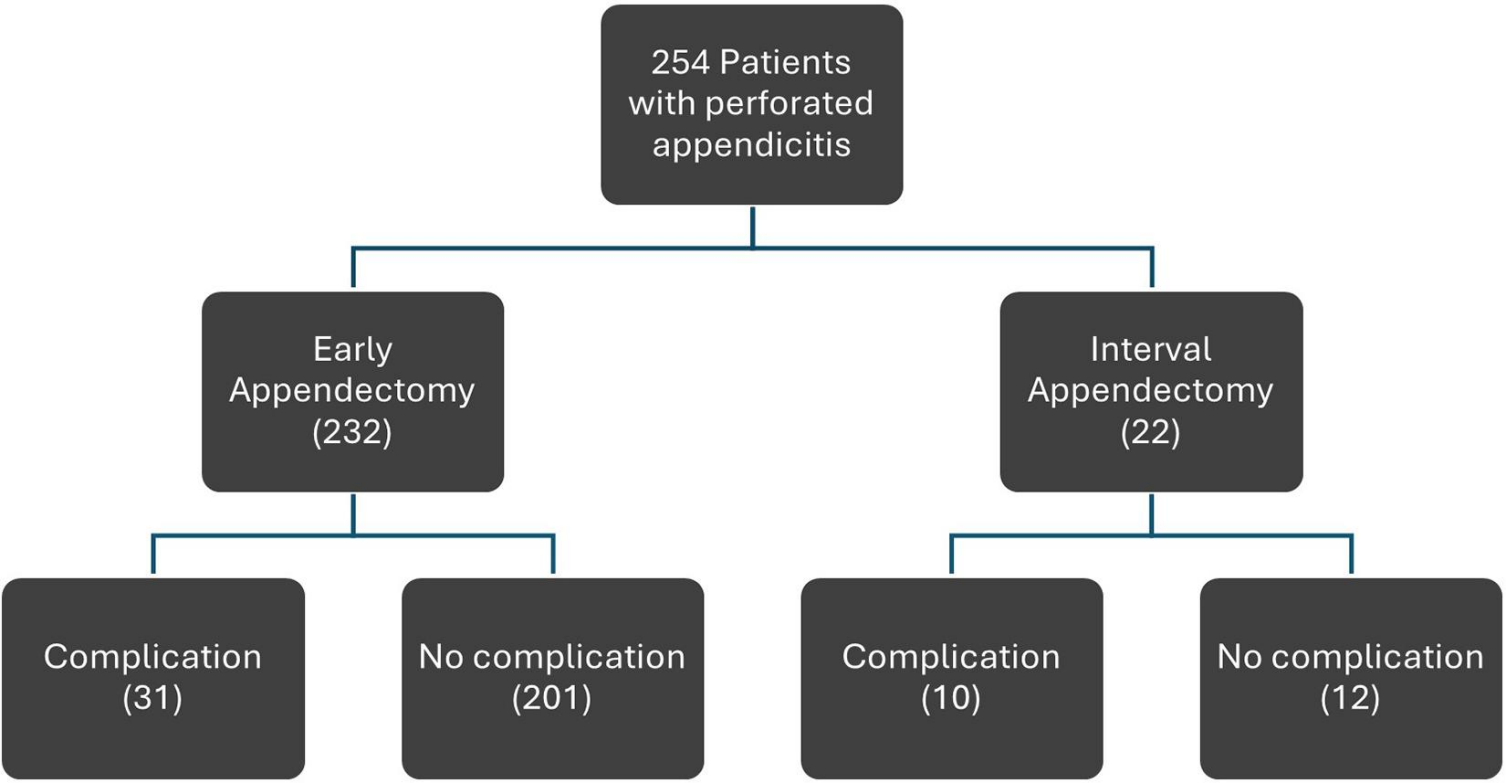
This flowchart illustrates patient distribution and clinical management pathways throughout the study.

A total of 1,017 patients were screened. Of these, 763 were excluded after detailed review due to the absence of complicated appendicitis, leaving 254 patients in the final study cohort (Figure 1).

### Variables and data collection

Baseline demographic variables including age and sex distribution are presented in Table 1, along with clinical and laboratory parameters (CRP, WBC, serum sodium, body temperature) measured at admission.

**Table 1:** Baseline characteristics. Overview of mean age, gender distribution, initial blood works (CRP, C-reactive protein; WBC, white blood cell count, sodium) and body temperature in both studied groups, EA, Early Appendectomy; and IA, Interval Appendectomy. SD standard deviation, MD mean difference, ^a^ mean, ^b^ unpaired *t*-test, ^c^ Fisher's exact test, d Mann–Whitney-U test.

Baseline characteristics	All *n* = 254	EA *n* = 232	IA *n* = 22	*p*-value	SD	MD
Age [y]	10.2	10.2	9.9	0.36^a^	4.1	0.3
Gender [m/f]	133/121	118/114	15/7	0.09^b^	0.5	103/107
CRP [mg/dL]	9.6	9.1	12.5	0.003^c^	8.1	3.4
WBC [G/L]	16.18	16.02	16.8	0.39^c^	5.7	0.8
Sodium [mmol/L]	136	136.1	134.3	0.94^c^	3.1	1.8
Temperature [C°]	37.8	37.8	38.1	0.17^c^	0.9	0.3

Of the 254, 232 patients (91.3%) underwent early appendectomy (EA), while 22 patients (8.7%) were treated with interval appendectomy (IA). Further, in the IA group, 12 of the 22 patients underwent CT-guided drainage during their initial course of treatment.

The mean age of the overall cohort was 10.2 ± 4.1 years. There were 133 male patients (52.4%) and 121 female patients (47.6%). In the EA group, the mean age was 10.2 ± 4.07 years, with 118 boys and 114 girls. In the IA group, the mean age was 9.9 ± 4.5 years, with 15 boys and 7 girls. There were no statistically significant differences between the groups regarding age or sex distribution.

The median length of hospital stay (LOH) was significantly shorter among patients undergoing EA compared to IA, with a median duration of 7 days (IQR 5–10 days) vs. 18 days (IQR 13–26), respectively (*p* < 0.001). This corresponded to a mean difference of 11 days (Table 2). Institutional treatment protocols defined an expected LOH of 7 days for EA and 9 days for IA (7 days during the initial admission and 2 days during the planned interval appendectomy). The difference between the observed and protocol-defined LOH remained significant (*p* < 0.001).

**Table 2:** Overview of the difference between the median average observed and median protocol-defined length of hospital stay (LOH) in early appendectomy (EA) and interval appendectomy (IA). *P*-values were calculated using the Mann–Whitney U test. Institutional treatment protocols defined an expected length of hospital stay of 7 days for early appendectomy and 9 days for interval appendectomy (7 days during the initial admission and 2 days during the planned interval appendectomy).

Outcome	All	Early Appendectomy (EA, *n* = 232)	Interval Appendectomy (IA, *n* = 22)	*p*-value*	Median Difference (EA–IA), days (95% CI)
Length of hospital stay, median (days)	8	7	18	<0.001	−11 (6 to 18)
Length of hospital stay > expected, median (days)	1	0	9	<0.001	−7.5 (4 to 16)

To assess factors influencing LOH apart from the surgical approach, we performed simple linear regression analyses for admission CRP, WBC count, serum sodium, body temperature, and patient age (Table 3). CRP at admission was not significantly associated with LOH in either the EA or IA cohorts (EA: *β* = 0.086, *p* = 0.174, *R*^2^ = 0.008; IA: *β* = 0.713, *p* = 0.091, *R*^2^ = 0.143). In contrast, WBC count emerged as a significant negative predictor of LOH in both groups (EA: *β* = –0.27, *p* = 0.004, *R*^2^ = 0.037; IA: *β* = –0.47, *p* = 0.031, *R*^2^ = 0.222). Patients with higher WBC values at admission experienced shorter hospital stays. Serum sodium and admission body temperature showed no significant relationships with LOH (sodium—EA: *β* = –0.27, *p* = 0.114, *R*^2^ = 0.012; IA: *β* = –0.73, *p* = 0.37, *R*^2^ = 0.054; temperature—EA: *β* = 0.55, *p* = 0.211, *R*^2^ = 0.007; IA: *β* = 1.19, *p* = 0.564, *R*^2^ = 0.020). Age demonstrated an inverse relationship with LOH in the EA cohort (*β* = –0.28, *p* = 0.022, *R*^2^ = 0.023). This effect was not observed in the IA cohort (*β* = 0.45, *p* = 0.55, *R*^2^ = 0.019).

**Table 3:** Simple linear regression analysis within the groups of early appendectomy (EA) and interval appendectomy (IA). Length of hospital stay as dependent variable, C-reactive protein (CRP), white blood cell count (WBC), serum sodium, body temperature and age.

Predictor (Admission Value)	EA: *β* (95% CI)	EA: p	EA: R²	IA: β (95% CI)	IA: p	IA: R²
CRP	0.086	0.174	0.008	0.713	0.091	0.143
WBC	−0.27	0.004	0.037	−0.47	0.031	0.222
Sodium	−0.27	0.114	0.012	−0.73	0.370	0.054
Temperature	0.55	0.211	0.007	1.19	0.564	0.020
Age	−0.28	0.022	0.023	0.45	0.550	0.019

### Predictors of length of hospitalization

To further explore factors influencing LOH, multiple linear regression analysis was performed. Among patients undergoing early appendectomy (EA), admission CRP emerged as an independent predictor of prolonged hospitalization (*β* = 0.117, *p* = 0.028) although the overall model demonstrated only modest explanatory power (R² = 0.072; *F*(5,184) = 2.874; *p* = 0.016). None of the other admission markers reached statistical significance in the EA cohort. In contrast, analysis of IA patients identified no significant predictors of LOH among the studied variables.

### Complications

Complications occurred in 13.8% of patients undergoing early appendectomy (32/232; 95% CI, 8.2–16.8%) and in 45.5% of those undergoing interval appendectomy (10/22; 95% CI, 26.3–66.2%). The absolute risk difference was −31.7% (95% CI, −50% to −14%) (Fisher's exact *p* < 0.001). The confidence interval around the complication rate in the interval appendectomy group was wide and included values approaching those observed in the early appendectomy group. Logistic regression analyses performed for CRP, WBC, serum sodium, body temperature, and age revealed no significant association between these variables and the occurrence of complications in either group.

The spectrum of complications (Table 4) was broadly similar in both groups, with intra-abdominal abscess, abdominal wall abscess, and wound infection being the most common in EA, and intra-abdominal abscess, recurrent symptoms, and wound infection in IA. However, severe complications including ileostomy creation, stump insufficiency, and ileocecal pole resection were observed exclusively in patients undergoing EA and were not reported in the IA group.

**Table 4:** Overview of all complications, sorted by occurrence in both groups early appendectomy (EA) and interval appendectomy (IA).

Complications	Clavien-Dindo-Classification	EA	IA	*p*-value
Total number of patients		232 (100%)	22 (100%)	
Readmission to hospital	level 1	21 (9,05%)	8 (36,36%)	<0,001
Intra-abdominal abscess	**level 3**	14 (6,03%)	3 (13,63%)	0,17
Repeat surgery	**level 3**	14 (6,03%)	3 (13,63%)	0,17
Free fluid	level1	8 (3,45%)	0 (0%)	
Abdominal wall abscess and umbilical abscess	**level 3**	7 (5,2%)	0 (0%)	0,45
Wound infection	level 2	5 (2,16%)	2 (9,1%)	
Abdominal wall abscess	**level 3**	5 (2,16%)	0 (0%)	
Wound dehiscence	level 1	4 (1,72%)	0 (0%)	
Sepsis	**level 4**	3 (1,3%)	1 (4,5%)	
Ileocecal resection	**level 4**	3 (1,3%)	0 (0%)	0,55
Paralytic ileus	**level 2–3**	3 (1,3%)	0 (0%)	0,55
Umbilical abscess	level 1	2 (0,86%)	0 (0%)	
Generalized peritonitis	level 2	2 (0,86%)	0 (0%)	
Ileostomy	**level 3**	2 (0,86%)	1 (4,5%)	0,14
Recurrent abdominal symptoms	level 1	1 (0,43%)	3 (13,63%)	
Umbilical hernia	**level 3**	1 (0,43%)	0 (0%)	
Stump insufficiency	**level 3**	1 (0,43%)	0 (0%)	0,65
Seroma formation	level 1	1 (0,43%)	0 (0%)	0,65
Terminal ileitis	level 2	1 (0,43%)	0 (0%)	0,65
Skin abscess	level 1	0 (0%)	1 (4,5%)	
Abdominal wall cellulitis	level 2	0 (0%)	1 (4,5%)	

The Clavien-Dindo Classification was applied and level 3 or higher was marked with bold values.

## Discussion

This study compared early (EA) and interval appendectomy (IA) in children with complicated appendicitis over a 12-year period. As expected, EA was associated with a significantly shorter hospital stay compared with IA. Overall complications occurred significantly more frequently in the IA group. However, severe complications, such as stump insufficiency, or those requiring ileostomy creation or ileocecal pole resection and thus carrying considerable implications for long-term morbidity and quality of life, were observed exclusively in the EA group. Although IA was associated with longer overall hospitalization and a higher rate of minor complications such as intra-abdominal abscess or recurrent symptoms, the complications encountered were generally less severe and could usually be managed without major reintervention.

In this study, early appendectomy was associated with a lower observed complication rate compared with interval appendectomy. However, complications in the early appendectomy group tended to be fewer but more severe, whereas interval appendectomy was associated with more frequent, generally less severe complications. Although the interval appendectomy group had a smaller sample size, resulting in wider confidence intervals, the data suggest that early appendectomy may reduce the overall number of complications. Importantly, the range of complication rates for interval appendectomy includes values that are not substantially higher than those observed for early appendectomy, indicating that while early appendectomy appears favorable, interval appendectomy is not clearly unsafe. These findings support individualized treatment decisions and highlight a central problem in the management of pediatric complicated appendicitis: whether to prioritize efficiency and reduced inpatient burden with EA, or to favor IA, which prolongs hospitalization but appears to carry a lower risk of harmful complications. While EA reduces the inpatient burden, the potential for severe adverse outcomes may limit its generalizability as the preferred standard of care. Our analysis of potential prognostic markers adds an additional layer to this decision-making process. The regression analysis highlights that CRP was the only independent predictor of prolonged LOH in EA, whereas no reliable prognostic markers were identified for IA. This indicates that systemic inflammation may prolong recovery in EA patients, but it also underlines that the inherent risks of EA are not easily predicted by routine laboratory tests at admission. Higher WBC ad admission was linked to shorter LOH, with the effect in the EA group explaining only a minor part of the variance in LOH, whereas the association in the IA group accounted for 1/5 of the variance, suggesting a more clinically meaningful impact.

Compared to the studies, both similar and different results are evident. As described in earlier studies by Nadler et al., Blakely et al. and Veeralakshmanan et al, our results also showed a shorter LOH in EA and fewer complications compared to IA (13–15), resulting in a significant reduction in morbidity (16). There were significantly fewer adverse events, such as unexpected readmissions, after EA (17). However, our study demonstrated that the complications in EA were more severe than in IA, drawing a contrast to the study of Huerta et al. which discovered a similar type of complication between both groups (3). IA therefore represented a safe treatment option, while there were more severe complications within the group of EA. These results confirm the observations of Duggan et al. and Bufo et al., which also describe IA as a safe and effective treatment option (18–20). It has been demonstrated previously in selected children, with reduced complication rates and shorter hospitalization (21, 22). It is possible that the severity of complications from surgery may result from acutely inflamed tissue. Other authors report no significant differences between EA and IA in patients with abscess formation (18). In contrast, the study by Vane et al. reports a shorter LOH and fewer complications in selected patient groups in IA (21). Our study did not identify any patient characteristics or parameters taken at admission in IA associated with shorter LOH and fewer complications. Munoz et al. were able to show that younger children benefit significantly from EA, while our study showed that there is an inverse correlation within in the group of EA in reference to LOH and age (23).

When viewed alongside existing literature, our results support a more individualized approach rather than universal application of EA. Several previous studies have demonstrated similar trade-offs, where EA offers efficiency but may expose patients to higher operative risk. In the pediatric population, where long-term sequelae are of particular concern, prioritizing safety may outweigh the advantage of reduced hospitalization.

Limitations of this study include its retrospective single-center design. Further, a key limitation of this study is the imbalance between patients undergoing early appendectomy (EA) and those managed with interval appendectomy (IA) since these cohorts may represent distinct clinical scenarios. Patients selected for IA may present with a localized abscess or inflammatory mass and a more prolonged disease course. It is important to emphasize that our study exclusively included patients with perforated appendicitis. Further, at our institution, management of complicated appendicitis follows an individualized approach rather than rigid protocolization, reflecting and contributing to the ongoing discussion in the literature regarding the optimal timing and therapeutic strategy for this condition. Both early and delayed surgical approaches are employed where clinically appropriate, reflecting the ongoing heterogeneity and controversy surrounding optimal management of complicated appendicitis as reported in the literature (24, 25). This approach may strengthen the external validity of our findings by reflecting real-world clinical conditions and enhancing their generalizability across diverse healthcare settings, although it may come at the expense of some degree of internal validity inherent to more tightly controlled study designs. Primary non-operative management is not routinely offered at our center. Variations in antibiotic regimens primarily reflected adjustments based on individual antibiograms, rather than treatment group assignment or institutional inconsistency.

Further, while severe complications were observed only in the EA group in this study, this finding should be interpreted with caution due to the relatively small sample size of the IA cohort and the overall low incidence of such events. The lack of severe complications in the IA group does not eliminate the possibility of risk. Because the absolute number of severe complications was low and the resulting confidence intervals are wide, comparisons are limited. Prospective, multicenter randomized studies are needed to better delineate which patients may benefit most from EA vs. IA, and whether laboratory or clinical markers can aid in risk stratification. Selection bias cannot be ruled out, since the groups were not randomized.

## Conclusion

Although early appendectomy significantly reduces hospital stay compared with interval appendectomy, it may carry a risk of severe complications not observed in the IA cohort in this study. Interval appendectomy, despite longer hospitalization and more frequent but less severe complications, may offer a safer overall approach for children with complicated appendicitis. Until reliable predictors of severe adverse events in EA are identified, IA should remain an important treatment option, and the choice of strategy should be individualized based on patient condition, surgeon experience, and family preference. These results underline the need for further prospective research aimed at identifying robust predictors of severe complications in EA.

### Clinical implications

Early appendectomy in children with complicated appendicitis shortens hospital stay but carries a risk of rare, severe complications.

Interval appendectomy prolongs hospitalization and leads to more minor complications but may appear safer with respect to major morbidity.

No reliable admission markers predict which children are at risk for severe adverse events following early appendectomy.

Treatment decisions should be individualized, balancing efficiency against safety, and incorporating patient condition, surgical expertise, and family preferences.

## Data Availability

The raw data supporting the conclusions of this article will be made available by the authors, without undue reservation.

## References

[1] Giuseppe Nigri, MD, PhD, FACS, FRCS. Gastrointestinal SURGICAL EMERGENCIES: American College of Surgeons International Relations Committee. Chicago: American College of Surgeons (2021).

[2] St. Peter SD Snyder CL. Operative management of appendicitis. Semin Pediatr Surg. (2016) 25(4):208–11. 10.1053/j.sempedsurg.2016.05.003 27521710

[3] Huerta CT Courel SC Ramsey WA Saberi RA Gilna GP Ribieras AJ Nationwide management of perforated pediatric appendicitis: interval versus same-admission appendectomy. J Pediatr Surg. (2023) 58(4):651–7. 10.1016/j.jpedsurg.2022.12.009 36641313

[4] Schweinitz Dv Ure B. Kinderchirurgie. Berlin, Heidelberg: Springer Berlin Heidelberg (2018).

[5] Stefanutti G Ghirardo V Gamba P. Inflammatory markers for acute appendicitis in children: are they helpful? J Pediatr Surg. (2007) 42(5):773–6. 10.1016/j.jpedsurg.2006.12.028 17502181

[6] Andersson REB. Meta-analysis of the clinical and laboratory diagnosis of appendicitis. Br J Surg. (2004) 91(1):28–37. 10.1002/bjs.4464 14716790

[7] Elgendy A Khirallah MG Elsawaf M Hassan HS Ghazaly M. Acute appendicitis in children: is preoperative hyponatremia a predictive factor of perforation/gangrene? A prospective study. Pediatr Surg Int. (2023) 39(1):281. 10.1007/s00383-023-05561-4 37817011 PMC10564656

[8] Pogorelić Z Domjanović J Jukić M Poklepović Peričić T. Acute appendicitis in children younger than five years of age: diagnostic challenge for pediatric surgeons. Surg Infect (Larchmt). (2020) 21(3):239–45. 10.1089/sur.2019.175 31618143

[9] Miyauchi H Okata Y Hatakeyama T Nakatani T Nakai Y Bitoh Y. Analysis of predictive factors for perforated appendicitis in children. Pediatr Int. (2020) 62(6):711–5. 10.1111/ped.14148 31957108

[10] Bansal S Banever GT Karrer FM Partrick DA. Appendicitis in children less than 5 years old: influence of age on presentation and outcome. Am J Surg. (2012) 204(6):1031–5; discussion 1035. 10.1016/j.amjsurg.2012.10.003 23231939

[11] Mostyka M Yantiss RK Chen Z Tseng-Chen Y. Interval appendectomy specimens. Arch Pathol Lab Med. (2022) 147(5):546–51. 10.5858/arpa.2021-0485-oa 36084245

[12] Pederiva F Bussani R Shafiei V Codrich D Guida E Schleef J. The histopathology of the appendix in children at interval appendectomy. Children (Basel). (2021) 8(9):811. 10.3390/children8090811 34572243 PMC8466409

[13] Nadler EP Reblock KK Vaughan KG Meza MP Ford HR Gaines BA. Predictors of outcome for children with perforated appendicitis initially treated with non-operative management. Surg Infect (Larchmt). (2004) 5(4):349–56. 10.1089/sur.2004.5.349 15744126

[14] Blakely ML Williams R Dassinger MS Eubanks JW Fischer P Huang EY Early vs interval appendectomy for children with perforated appendicitis. Arch Surg. (2011) 146(6):660–5. 10.1001/archsurg.2011.6 21339413

[15] Veeralakshmanan P Ackah J Panahi P Ibrahim R Coleman M. Early versus interval appendicectomy for localised perforated appendicitis in children: a best evidence review. Ann Med Surg (Lond). (2020) 59:161–4. 10.1016/j.amsu.2020.09.028 33082944 PMC7551639

[16] Bonadio W Rebillot K Ukwuoma O Saracino C Iskhakov A. Management of pediatric perforated appendicitis: comparing outcomes using early appendectomy versus solely medical management. Pediatr Infect Dis J. (2017) 36(10):937–41. 10.1097/INF.0000000000001025 26669739

[17] Myers AL Williams RF Giles K Waters TM Eubanks JW Hixson SD Hospital cost analysis of a prospective, randomized trial of early vs interval appendectomy for perforated appendicitis in children. J Am Coll Surg. (2012) 214(4):427–34; discussion 434-5. 10.1016/j.jamcollsurg.2011.12.026 22342789

[18] Duggan EM Marshall AP Weaver KL St Peter SD Tice J Wang L A systematic review and individual patient data meta-analysis of published randomized clinical trials comparing early versus interval appendectomy for children with perforated appendicitis. Pediatr Surg Int. (2016) 32(7):649–55. 10.1007/s00383-016-3897-y 27161128

[19] Bufo AJ Shah RS Li MH Cyr NA Hollabaugh RS Hixson SD Interval appendectomy for perforated appendicitis in children. J Laparoendosc Adv Surg Tech A. (1998) 8(4):209–14. 10.1089/lap.1998.8.209 9755912

[20] Gonzalez DO Deans KJ Minneci PC. Role of non-operative management in pediatric appendicitis. Semin Pediatr Surg. (2016) 25(4):204–7. 10.1053/j.sempedsurg.2016.05.002 27521709

[21] Vane DW Fernandez N. Role of interval appendectomy in the management of complicated appendicitis in children. World J Surg. (2006) 30(1):51–4. 10.1007/s00268-005-7946-2 16369706

[22] Henry MCW Gollin G Islam S Sylvester K Walker A Silverman BL Matched analysis of nonoperative management vs immediate appendectomy for perforated appendicitis. J Pediatr Surg. (2007) 42(1):19–23; discussion 23–4. 10.1016/j.jpedsurg.2006.09.005 17208535

[23] Munoz A Hazboun R Vannix I Pepper V Crane T Tagge E Young children with perforated appendicitis benefit from prompt appendectomy. J Pediatr Surg. (2019) 54(9):1809–14. 10.1016/j.jpedsurg.2018.10.107 30638663

[24] van den Boom AL de Wijkerslooth EML Mauff KAL Dawson I van Rossem CC Toorenvliet BR Interobserver variability in the classification of appendicitis during laparoscopy. Br J Surg. (2018) 105(8):1014–9. 10.1002/bjs.10837 29663311 PMC6033013

[25] Badru F Piening N Munoz Abraham AS Osei H Greenspon J Chatoorgoon K Abscess and symptoms duration upon presentation should guide decision algorithms for early versus interval appendectomy in children. Pediatr Neonatol. (2019) 60(5):530–6. 10.1016/j.pedneo.2019.01.005 30737113

